# Ethyl 2-(quinolin-8-yl­oxy)acetate monohydrate

**DOI:** 10.1107/S1600536813008106

**Published:** 2013-03-28

**Authors:** Mohan Kumar, C. Mallikarjunaswamy, M. A. Sridhar, D. G. Bhadregowda, Kamini Kapoor, Vivek K. Gupta, Rajni Kant

**Affiliations:** a Department of Studies in Physics, Manasagangotri, University of Mysore, Mysore 570 006, India; b Department of Chemistry, Yuvarajas College, University of Mysore, Mysore 570005, India; c X-ray Crystallography Laboratory, Post-Graduate Department of Physics & Electronics, University of Jammu, Jammu Tawi 180 006, India

## Abstract

In the title compound, C_13_H_13_NO_3_·H_2_O, the dihedral angle between the ethyl ester group [C—C—O—C(=O); maximum deviation = 0.003 (2) Å] and the quinoline ring system is 7.94 (12)°. The water solvent mol­ecule is linked to the title mol­ecule *via* O—H⋯O and O—H⋯N hydrogen bonds. In the crystal, mol­ecules are linked by C—H⋯O hydrogen bonds, forming chains propagating along [100].

## Related literature

For related structures see: Sarveswari *et al.* (2010 ▶); Ukrainets *et al.* (2009 ▶). For bond-length data, see: Allen *et al.* (1987 ▶).
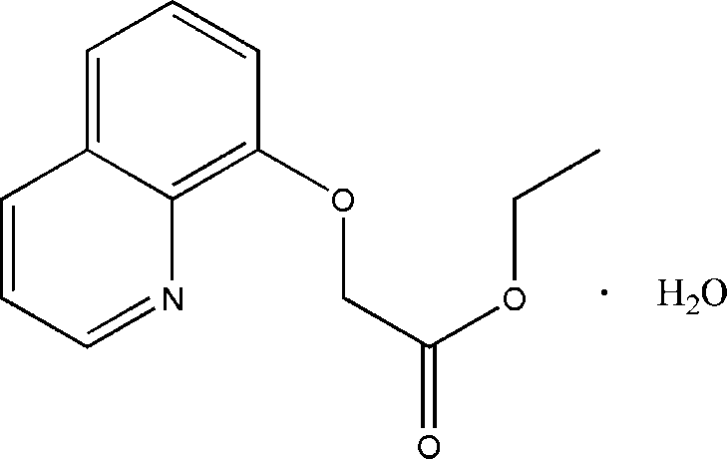


## Experimental

### 

#### Crystal data

C_13_H_13_NO_3_·H_2_O
*M* *_r_* = 249.26
Monoclinic, 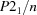

*a* = 6.9562 (4) Å
*b* = 17.5050 (9) Å
*c* = 10.5304 (6) Å
β = 100.124 (5)°
*V* = 1262.30 (12) Å^3^
*Z* = 4
Mo *K*α radiation
μ = 0.10 mm^−1^
*T* = 293 K
0.3 × 0.2 × 0.2 mm

#### Data collection

Oxford Diffraction Xcalibur Sapphire3 diffractometer
Absorption correction: multi-scan (*CrysAlis PRO*; Oxford Diffraction, 2010 ▶) *T*_min_ = 0.715, *T*_max_ = 1.000
9808 measured reflections
2478 independent reflections
1448 reflections with *I* > 2σ(*I*)
*R*_int_ = 0.051

#### Refinement

*R*[*F* ^2^ > 2σ(*F* ^2^)] = 0.053
*wR*(*F* ^2^) = 0.123
*S* = 1.02
2478 reflections
172 parameters
H atoms treated by a mixture of independent and constrained refinement
Δρ_max_ = 0.14 e Å^−3^
Δρ_min_ = −0.19 e Å^−3^

### 

Data collection: *CrysAlis PRO* (Oxford Diffraction, 2010 ▶); cell refinement: *CrysAlis PRO*; data reduction: *CrysAlis RED* (Oxford Diffraction, 2010 ▶); program(s) used to solve structure: *SHELXS97* (Sheldrick, 2008 ▶); program(s) used to refine structure: *SHELXL97* (Sheldrick, 2008 ▶); molecular graphics: *ORTEP-3 for Windows* (Farrugia, 2012 ▶); software used to prepare material for publication: *PLATON* (Spek, 2009 ▶).

## Supplementary Material

Crystal structure: contains datablock(s) I, global. DOI: 10.1107/S1600536813008106/su2575sup1.cif

Structure factors: contains datablock(s) I. DOI: 10.1107/S1600536813008106/su2575Isup2.hkl

Supplementary material file. DOI: 10.1107/S1600536813008106/su2575Isup3.cml

Additional supplementary materials:  crystallographic information; 3D view; checkCIF report

## Figures and Tables

**Table 1:** Hydrogen-bond geometry (Å, °)

*D*—H⋯*A*	*D*—H	H⋯*A*	*D*⋯*A*	*D*—H⋯*A*
O1*W*—H1*W*⋯O5	0.85 (3)	2.06 (3)	2.907 (3)	172
O1*W*—H2*W*⋯N16	0.92 (3)	1.96 (3)	2.875 (3)	174
C6—H6*A*⋯O1*W* ^i^	0.97	2.43	3.388 (3)	170

Symmetry code: (i) 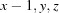.

**Fig. 1.**
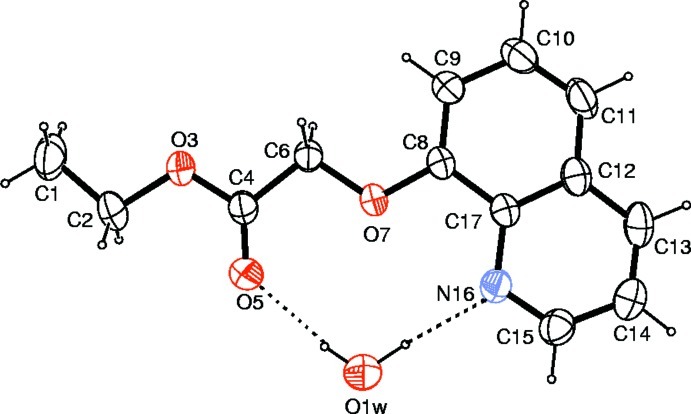
A view of the molecular structure of the title compound, with atom labelling. Displacement ellipsoids are drawn at the 40% probability level. The O-H···O and O-H···N hydrogen bonds are shown as dashed lines (see Table for details).

**Fig. 2.**
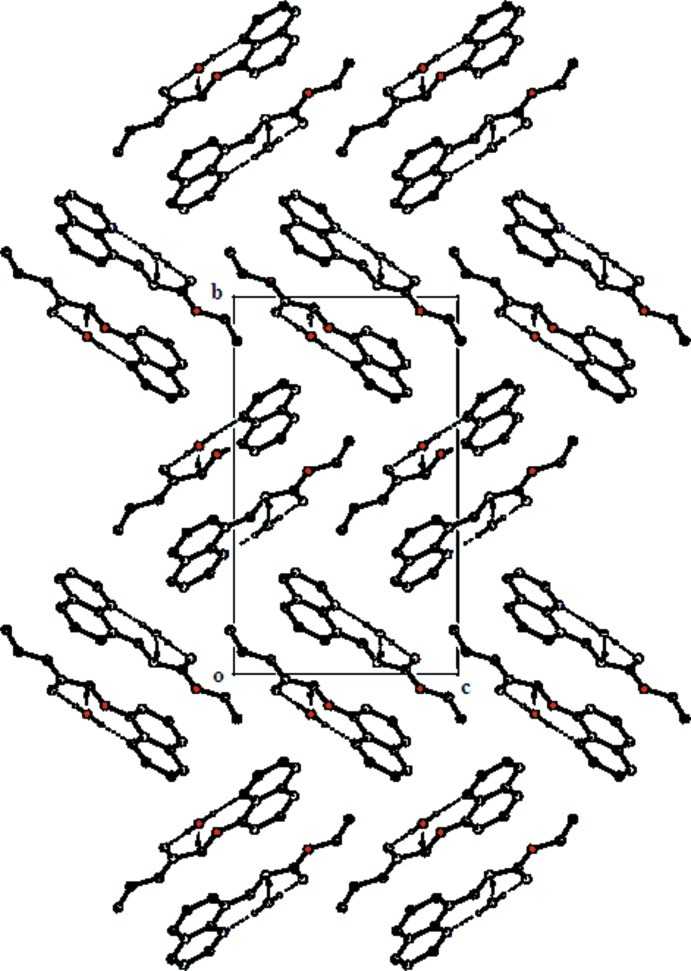
The crystal packing of the title compound view along the a axis, showing the O—H···O, O—H···N and C-H···O hydrogen bonds as dashed lines (see Table 1 for details).

## supplementary crystallographic information

### Comment

In the title molecule, Fig. 1, the bond lengths (Allen *et al.*, 1987) and angles have normal values and are comparable with those reported for similar structures (Sarveswari *et al.*, 2010; Ukrainets *et al.*, 2009). The dihedral angle between the ethyl ester group (C1/C2/O3/C4/O5) and the quinoline (C8—C15/N16/C17) ring system is 7.94 (12)°. The solvent water molecule is linked to the title molecule via O-H···O and O-H···N hydrogen bonds (Fig. 1 and Table 1).

In the crystal, molecules are linked by C—H···O hydrogen bonds (Table 1) forming chains propagating along the a axis direction (Fig. 2).

### Experimental

A mixture of 8-hydroxy quinoline(0.01 mol) and ethyl chloroacetate (0.015 mol) in the presence of dry acetone (50 ml) and anhydrous potassium carbonate (0.015 mol) was refluxed for 8 h. The residual mass was triturated with cold water to remove potassium carbonate and extracted with ether (30 ml). The ether layer was washed with 10% sodium hydroxide solution (350 ml) followed by water (330 ml) and then dried over anhydrous sodium sulfate and evaporated to dryness. The compound was purified by successive recrystallizations from ethanol yielding colourless block-like crystals (Yield 90%, m.p. 350–352 K).

### Refinement

The water molecule H atoms were located in a difference Fourier map and freely refined. The C-bound H atoms were positioned geometrically and treated as riding atoms: C—H distances of 0.93–0.97 Å with *U*_iso_(H) = 1.5*U*_eq_(methyl C) and = 1.2*U*_eq_(C) for other H atoms.

### Crystal data

**Table:**

C_13_H_13_NO_3_·H_2_O	*F*(000) = 528
*M**_r_* = 249.26	*D*_x_ = 1.312 Mg m^−^^3^
Monoclinic, *P*2_1_/*n*	Mo *K*α radiation, λ = 0.71073 Å
Hall symbol: -P 2yn	Cell parameters from 3127 reflections
*a* = 6.9562 (4) Å	θ = 3.5–29.0°
*b* = 17.5050 (9) Å	µ = 0.10 mm^−^^1^
*c* = 10.5304 (6) Å	*T* = 293 K
β = 100.124 (5)°	Block, colourless
*V* = 1262.30 (12) Å^3^	0.3 × 0.2 × 0.2 mm
*Z* = 4	

### Data collection

**Table:**

Oxford Diffraction Xcalibur Sapphire3 diffractometer	2478 independent reflections
Radiation source: fine-focus sealed tube	1448 reflections with *I* > 2σ(*I*)
Graphite monochromator	*R*_int_ = 0.051
Detector resolution: 16.1049 pixels mm^-1^	θ_max_ = 26.0°, θ_min_ = 3.5°
ω scans	*h* = −8→8
Absorption correction: multi-scan (*CrysAlis PRO*; Oxford Diffraction, 2010)	*k* = −21→21
*T*_min_ = 0.715, *T*_max_ = 1.000	*l* = −11→12
9808 measured reflections	

### Refinement

**Table:**

Refinement on *F*^2^	Primary atom site location: structure-invariant direct methods
Least-squares matrix: full	Secondary atom site location: difference Fourier map
*R*[*F*^2^ > 2σ(*F*^2^)] = 0.053	Hydrogen site location: inferred from neighbouring sites
*wR*(*F*^2^) = 0.123	H atoms treated by a mixture of independent and constrained refinement
*S* = 1.02	*w* = 1/[σ^2^(*F*_o_^2^) + (0.0382*P*)^2^ + 0.1537*P*] where *P* = (*F*_o_^2^ + 2*F*_c_^2^)/3
2478 reflections	(Δ/σ)_max_ = 0.001
172 parameters	Δρ_max_ = 0.14 e Å^−^^3^
0 restraints	Δρ_min_ = −0.19 e Å^−^^3^

### Special details

**Table:**

Geometry. All e.s.d.'s (except the e.s.d. in the dihedral angle between two l.s. planes) are estimated using the full covariance matrix. The cell e.s.d.'s are taken into account individually in the estimation of e.s.d.'s in distances, angles and torsion angles; correlations between e.s.d.'s in cell parameters are only used when they are defined by crystal symmetry. An approximate (isotropic) treatment of cell e.s.d.'s is used for estimating e.s.d.'s involving l.s. planes.
Refinement. Refinement of *F*^2^ against ALL reflections. The weighted *R*-factor *wR* and goodness of fit *S* are based on *F*^2^, conventional *R*-factors *R* are based on *F*, with *F* set to zero for negative *F*^2^. The threshold expression of *F*^2^ > σ(*F*^2^) is used only for calculating *R*-factors(gt) *etc*. and is not relevant to the choice of reflections for refinement. *R*-factors based on *F*^2^ are statistically about twice as large as those based on *F*, and *R*- factors based on ALL data will be even larger.

### Fractional atomic coordinates and isotropic or equivalent isotropic displacement parameters (Å^2^)

**Table:**

	*x*	*y*	*z*	*U*_iso_*/*U*_eq_	
C1	0.1064 (4)	−0.11734 (16)	−0.0152 (2)	0.0746 (9)	
H1A	0.1136	−0.1608	0.0410	0.112*	
H1B	0.1291	−0.1334	−0.0985	0.112*	
H1C	−0.0208	−0.0946	−0.0237	0.112*	
C2	0.2574 (4)	−0.06026 (15)	0.0399 (2)	0.0605 (7)	
H2A	0.3868	−0.0825	0.0481	0.073*	
H2B	0.2511	−0.0160	−0.0160	0.073*	
O3	0.2179 (2)	−0.03807 (9)	0.16644 (14)	0.0527 (5)	
C4	0.3381 (3)	0.01324 (13)	0.2305 (2)	0.0431 (6)	
O5	0.4722 (3)	0.04156 (11)	0.19103 (16)	0.0671 (6)	
C6	0.2763 (3)	0.03087 (12)	0.35690 (19)	0.0407 (6)	
H6A	0.1472	0.0537	0.3421	0.049*	
H6B	0.2717	−0.0157	0.4062	0.049*	
O7	0.4143 (2)	0.08241 (8)	0.42563 (13)	0.0440 (4)	
C8	0.3737 (3)	0.11137 (12)	0.5390 (2)	0.0374 (5)	
C9	0.2176 (3)	0.09077 (14)	0.5928 (2)	0.0501 (6)	
H9	0.1299	0.0544	0.5529	0.060*	
C10	0.1893 (4)	0.12463 (16)	0.7087 (2)	0.0628 (7)	
H10	0.0824	0.1100	0.7451	0.075*	
C11	0.3136 (4)	0.17809 (16)	0.7690 (2)	0.0630 (8)	
H11	0.2902	0.2006	0.8449	0.076*	
C12	0.4787 (4)	0.19961 (13)	0.7166 (2)	0.0471 (6)	
C13	0.6180 (4)	0.25321 (14)	0.7755 (2)	0.0595 (7)	
H13	0.6003	0.2777	0.8510	0.071*	
C14	0.7760 (4)	0.26897 (14)	0.7230 (2)	0.0582 (7)	
H14	0.8698	0.3035	0.7620	0.070*	
C15	0.7960 (4)	0.23238 (13)	0.6085 (2)	0.0538 (7)	
H15	0.9056	0.2441	0.5728	0.065*	
N16	0.6718 (3)	0.18260 (11)	0.54715 (17)	0.0466 (5)	
C17	0.5122 (3)	0.16581 (12)	0.6004 (2)	0.0383 (5)	
O1W	0.8236 (3)	0.10488 (13)	0.3454 (2)	0.0723 (6)	
H2W	0.770 (5)	0.1318 (18)	0.406 (3)	0.123 (14)*	
H1W	0.728 (5)	0.0844 (18)	0.295 (3)	0.109 (13)*	

### Atomic displacement parameters (Å^2^)

**Table:**

	*U*^11^	*U*^22^	*U*^33^	*U*^12^	*U*^13^	*U*^23^
C1	0.099 (2)	0.0701 (19)	0.0512 (16)	−0.0165 (17)	0.0026 (16)	−0.0184 (14)
C2	0.0763 (19)	0.0683 (18)	0.0396 (14)	−0.0050 (15)	0.0171 (13)	−0.0153 (13)
O3	0.0602 (11)	0.0590 (11)	0.0403 (9)	−0.0135 (9)	0.0124 (8)	−0.0143 (8)
C4	0.0463 (14)	0.0449 (14)	0.0378 (13)	0.0001 (12)	0.0067 (11)	−0.0053 (11)
O5	0.0646 (12)	0.0882 (14)	0.0534 (11)	−0.0269 (11)	0.0243 (9)	−0.0198 (10)
C6	0.0409 (13)	0.0417 (13)	0.0394 (12)	−0.0017 (10)	0.0065 (10)	−0.0068 (10)
O7	0.0484 (9)	0.0490 (10)	0.0359 (8)	−0.0061 (8)	0.0110 (7)	−0.0102 (7)
C8	0.0442 (13)	0.0353 (12)	0.0327 (12)	0.0049 (10)	0.0067 (10)	−0.0018 (10)
C9	0.0499 (15)	0.0598 (17)	0.0424 (14)	−0.0055 (13)	0.0129 (12)	−0.0047 (12)
C10	0.0585 (17)	0.083 (2)	0.0517 (16)	−0.0014 (15)	0.0234 (13)	−0.0074 (15)
C11	0.0686 (18)	0.079 (2)	0.0443 (15)	0.0070 (16)	0.0184 (14)	−0.0182 (14)
C12	0.0577 (15)	0.0410 (14)	0.0413 (13)	0.0083 (12)	0.0054 (12)	−0.0041 (11)
C13	0.0715 (19)	0.0504 (16)	0.0531 (15)	0.0103 (14)	0.0015 (14)	−0.0194 (13)
C14	0.0637 (18)	0.0478 (16)	0.0590 (17)	−0.0028 (13)	−0.0003 (14)	−0.0094 (13)
C15	0.0588 (16)	0.0491 (15)	0.0524 (15)	−0.0068 (13)	0.0073 (13)	0.0025 (13)
N16	0.0512 (12)	0.0438 (12)	0.0446 (11)	−0.0055 (10)	0.0079 (10)	−0.0040 (9)
C17	0.0450 (13)	0.0358 (13)	0.0335 (12)	0.0054 (11)	0.0051 (10)	0.0010 (10)
O1W	0.0525 (12)	0.0896 (16)	0.0786 (15)	−0.0139 (11)	0.0217 (11)	−0.0260 (13)

### Geometric parameters (Å, º)

**Table:**

C1—C2	1.492 (3)	C9—H9	0.9300
C1—H1A	0.9600	C10—C11	1.354 (3)
C1—H1B	0.9600	C10—H10	0.9300
C1—H1C	0.9600	C11—C12	1.410 (3)
C2—O3	1.460 (3)	C11—H11	0.9300
C2—H2A	0.9700	C12—C13	1.412 (3)
C2—H2B	0.9700	C12—C17	1.414 (3)
O3—C4	1.328 (2)	C13—C14	1.344 (3)
C4—O5	1.194 (3)	C13—H13	0.9300
C4—C6	1.501 (3)	C14—C15	1.393 (3)
C6—O7	1.420 (2)	C14—H14	0.9300
C6—H6A	0.9700	C15—N16	1.314 (3)
C6—H6B	0.9700	C15—H15	0.9300
O7—C8	1.371 (2)	N16—C17	1.361 (3)
C8—C9	1.359 (3)	O1W—H2W	0.93 (4)
C8—C17	1.427 (3)	O1W—H1W	0.85 (4)
C9—C10	1.401 (3)		
			
C2—C1—H1A	109.5	C8—C9—C10	119.7 (2)
C2—C1—H1B	109.5	C8—C9—H9	120.1
H1A—C1—H1B	109.5	C10—C9—H9	120.1
C2—C1—H1C	109.5	C11—C10—C9	121.7 (2)
H1A—C1—H1C	109.5	C11—C10—H10	119.2
H1B—C1—H1C	109.5	C9—C10—H10	119.2
O3—C2—C1	107.5 (2)	C10—C11—C12	119.9 (2)
O3—C2—H2A	110.2	C10—C11—H11	120.1
C1—C2—H2A	110.2	C12—C11—H11	120.1
O3—C2—H2B	110.2	C11—C12—C13	123.3 (2)
C1—C2—H2B	110.2	C11—C12—C17	119.8 (2)
H2A—C2—H2B	108.5	C13—C12—C17	117.0 (2)
C4—O3—C2	116.17 (19)	C14—C13—C12	120.2 (2)
O5—C4—O3	124.4 (2)	C14—C13—H13	119.9
O5—C4—C6	125.9 (2)	C12—C13—H13	119.9
O3—C4—C6	109.7 (2)	C13—C14—C15	118.4 (2)
O7—C6—C4	108.00 (18)	C13—C14—H14	120.8
O7—C6—H6A	110.1	C15—C14—H14	120.8
C4—C6—H6A	110.1	N16—C15—C14	125.0 (3)
O7—C6—H6B	110.1	N16—C15—H15	117.5
C4—C6—H6B	110.1	C14—C15—H15	117.5
H6A—C6—H6B	108.4	C15—N16—C17	117.0 (2)
C8—O7—C6	117.01 (17)	N16—C17—C12	122.4 (2)
C9—C8—O7	124.6 (2)	N16—C17—C8	119.47 (19)
C9—C8—C17	120.9 (2)	C12—C17—C8	118.1 (2)
O7—C8—C17	114.56 (19)	H2W—O1W—H1W	107 (3)
			
C1—C2—O3—C4	−179.6 (2)	C17—C12—C13—C14	−1.4 (3)
C2—O3—C4—O5	0.3 (3)	C12—C13—C14—C15	1.2 (4)
C2—O3—C4—C6	179.04 (19)	C13—C14—C15—N16	−0.4 (4)
O5—C4—C6—O7	−4.6 (3)	C14—C15—N16—C17	−0.1 (3)
O3—C4—C6—O7	176.69 (16)	C15—N16—C17—C12	−0.1 (3)
C4—C6—O7—C8	173.95 (17)	C15—N16—C17—C8	−179.14 (19)
C6—O7—C8—C9	4.0 (3)	C11—C12—C17—N16	−178.0 (2)
C6—O7—C8—C17	−176.90 (17)	C13—C12—C17—N16	0.9 (3)
O7—C8—C9—C10	−179.4 (2)	C11—C12—C17—C8	1.0 (3)
C17—C8—C9—C10	1.5 (3)	C13—C12—C17—C8	179.91 (19)
C8—C9—C10—C11	0.3 (4)	C9—C8—C17—N16	177.0 (2)
C9—C10—C11—C12	−1.4 (4)	O7—C8—C17—N16	−2.2 (3)
C10—C11—C12—C13	−178.1 (2)	C9—C8—C17—C12	−2.1 (3)
C10—C11—C12—C17	0.7 (4)	O7—C8—C17—C12	178.72 (18)
C11—C12—C13—C14	177.4 (2)		

### Hydrogen-bond geometry (Å, º)

**Table:**

*D*—H···*A*	*D*—H	H···*A*	*D*···*A*	*D*—H···*A*
O1*W*—H1*W*···O5	0.85 (3)	2.06 (3)	2.907 (3)	172
O1*W*—H2*W*···N16	0.92 (3)	1.96 (3)	2.875 (3)	174
C6—H6*A*···O1*W*^i^	0.97	2.43	3.388 (3)	170

Symmetry code: (i) *x*−1, *y*, *z*.
